# Cytokine Signature Associated with Disease Severity in Dengue

**DOI:** 10.3390/v11010034

**Published:** 2019-01-08

**Authors:** A. Raj Kumar Patro, Sriprasad Mohanty, Birendra K. Prusty, Diwakar K. Singh, Sagar Gaikwad, Tanuja Saswat, Soma Chattopadhyay, Bidyut K. Das, Rina Tripathy, Balachandran Ravindran

**Affiliations:** 1 Infectious Disease Biology Group, Institute of Life Sciences, Bhubaneswar 751023, India; drbirendraprusty@gmail.com (B.K.P.); diwakarcdri@gmail.com (D.K.S.); Sagargaikwad2011@gmail.com (S.G.); tanusaswat@gmail.com (T.S.); soma@ils.res.in (S.C.); balaravi@ils.res.in (B.R.); 2 Departments of Medicine, SCB Medical College & Hospital, Cuttack 753007, India; drsriprasad@gmail.com (S.M.); bidyutdas@hotmail.com (B.K.D.); 3 Department of Biochemistry, SCB Medical College & Hospital, Cuttack 753007, India; rinatripathy@gmail.com

**Keywords:** Arbovirus, Dengue, Cytokine, Luminex-bead assay, Inflammation, Severe dengue

## Abstract

Dengue is the most rapidly spreading viral disease transmitted by the bite of infected *Aedes* mosquitos. The pathogenesis of dengue is still unclear; although host immune responses and virus serotypes have been proposed to contribute to disease severity. In this study, we examined the circulating dengue virus (DENV) and measured plasma levels of inflammatory mediators. Ninety-eight patients during a dengue outbreak in eastern India in 2016 were included in the study. The presence of DENV was demonstrated by detecting NS1 antigen; IgM capture ELISA and serotypes were discriminated by type-specific RT-PCR and/or sequencing. Plasma samples were assayed for 41-plex cytokine/chemokines using multiplex Luminex assay. Eighty-five (87%) samples were positive by NS1/IgM capture ELISA/RT-PCR. All four serotypes of DENV were detected in this outbreak, with DENV-2 as the predominant type, seen in 55% of cases. Mixed infections were seen in 39% of subjects. Among the host inflammatory biomarkers, GM-CSF, IFN-γ, IL-10, IL-15, IL-8, MCP-1, IL-6, MIP-1β, and TNF-α levels were significantly increased in dengue with and without warning signs, in severe dengue patients in comparison to healthy controls. Four cytokines IFN-γ, GM-CSF, IL-10, and MIP-1β correlated significantly with disease severity and could serve as potential predictor for disease severity. Information on the host biomarkers and the dengue serotype may help guide in optimizing effective intervention strategies.

## 1. Introduction

Dengue virus transmitted by *Aedes* mosquito, is an increasing global problem, with an estimate of 390 million infections per year and about 3.6 billion people at risk of dengue [1]. Infection with the dengue virus (DENV) results in a spectrum of clinical manifestations ranging from asymptomatic infection, self-limiting, dengue fever (DF) with or without warning signs, or to life threatening dengue (SD). Patients with dengue virus infections present with fever, headache, fatigue, nausea, chills, joint pain, and dizziness. In a cohort of patients, dengue infections lead to life threatening severe dengue characterized by vascular leakage leading to shock, internal hemorrhage, and organ impairment, which results in death if untreated. The detailed pathogenesis and mechanisms that led to severe clinical manifestations of dengue are currently not clear.

Four antigenically related but distinct serotypes of this virus have been reported, described as DENV-1, DENV-2, DENV-3, and DENV-4. The dengue virus is a positive strand RNA genome of 10.7 kb nucleotides, which encodes three structural (capsid, membrane, and envelope) and seven non-structural (NS1, NS2A, NS2B, NS3, NS4A, NS4B, and NS5) proteins [2]. Infection with one of the four viral serotypes confers protective immunity against re-infection to only the same serotype, while subsequent infections with other serotypes results in severe dengue through an antibody-dependent enhancement (ADE) leading to a cytokine storm [3,4,5]. However, a recent study by Waggoner et al., in 2016, reported homotypic dengue reinfection in four patients among 29 repeat DENV infections in an ongoing pediatric cohort study in Nicaragua [6] suggesting that infection to DENV does not provide lifelong immunity and a person can be infected with the same virus. Following infection with DENV, primary responses to viruses are mediated by the innate arm of the immune system, eliciting production of inflammatory and antiviral molecules. An exacerbated host immune response marked by antibody-dependent enhancement plays an important role in development of severe dengue [7,8,9]. The host immune response has been proposed to play a major role in pathogenesis of severe dengue. The mismatch of capillary permeability and the virus burden has resulted in a debate between the direct role of virus mediated action on the vascular epithelium and host immune response to the viral infection leading to the pathology. Further, Rothman et al., suggested that immunopathogenesis of severe dengue occurs in patients by antibody dependent enhancement (ADE) of dengue disease severity [4]. In all these scenarios, production of soluble inflammatory mediators is key to pathogenesis and supports the hypothesis of cytokine storm in ADE. However, studies on the multiple cytokines in a cohort of clinical specimens are limited in literature. Excessive production of pro-inflammatory cytokines (e.g. TNF-α, IFN-γ etc) drives progressive vascular leakage, leading to poor organ perfusion in patients with dengue infection [10,11]. On the other hand, levels of immunosuppressive cytokines such as IL-10 decrease during the critical phase; in contrast, inflammatory cytokines tend to increase. These complex interaction networks of several cytokines with negative and positive feedback mechanisms regulate pathogenesis of dengue, and their fine tuning determines the disease outcome. Although increased levels of some of the host pro-inflammatory molecules and vascular permeability in endothelial cells in dengue-infected patients have been reported in limited studies [4,10,12,13], a comprehensive study of many host response mediators involved in immune enhanced disease leading to hemorrhagic manifestations is yet to be undertaken. 

The availability of high throughput Luminex based cytokine bead assays (xMap cytokine bead array) allows the identification of the key markers of inflammation associated with disease severity [14]. Insights into these biomarkers of disease severity could also offer rational intervention strategies using antagonists of specific cytokines.

Recent development of a dengue vaccine, Dengvaxia (WHO, 2017) [15] has opened up new avenues of hope for reducing the disease burden. Because of the geographical preponderance of this virus, there are limited data on the circulating strains in India. More critically, data on whether or not serotype specific host responses are associated with disease severity is not available. Since the vaccine is likely to be available in near future, it is imperative to examine the relative distribution of the circulating dengue serotypes. The complex interactions of viral genetics and host immune determinants contribute to disease severity in Dengue. The current study was carried out to examine levels of 41 host inflammatory molecules and their correlation with disease severity by multiplex bead assay with the objective to identify biomarkers that could be used for devising interventional strategies.

## 2. Methods

### 2.1. Study Population

From May 2016 to December 2016, 98 subjects presented at the S.C.B. Medical College & Hospital, Cuttack, Odisha, India, were enrolled in the study (Figure S1). The subjects originated from four different states: Odisha, Telangana, West Bengal, and Jharkhand, of India. Patients with high fever, nausea, joint pain, dizziness, shock-like state, rash, drop in blood pressure and amplification of primary symptoms, vascular system damage and bleeding, consistent with WHO criteria 2009 [16,17], were included in the study. Blood samples were collected in both EDTA vacutainers to get plasma clotted blood and in serum tube with clot activator for separation of serum samples (4 ml in a K2 EDTA anticoagulant vacutainer and 4 ml in a Serum tube with clot activator, BD Biosciences) and immediately transported in ice to the laboratory. Plasma samples were used for cytokine assay by Luminex system and sera were used in ELISA and for viral RNA isolation. The samples were aliquoted and immediately processed for the experiment and the rest stored at −80 °C for further use. Age and sex matched healthy control subjects, mostly institutional volunteers and disease free persons accompanying patients in SCB Medical College, Cuttack, India, were included in the study. Institutional Human Ethic Committee Review Board approval was obtained at each study site, from Institute Human Ethics Review Committee of S.C.B. Medical College & Hospital and Institute of Life Sciences, India, and blood samples were collected after informed written consent was taken. 

### 2.2. Detection of Dengue Virus

Detection of the Dengue NS1 antigen and IgG on serum samples were done using BeneSphera Dengue Test Kit (Avantor, PA, USA,) according to manufacturer’s instructions. Qualitative detection of IgM antibodies to dengue antigens for recent infection in serum samples were performed using the Panbio® Dengue IgM Capture ELISA kit (01PE20, 96 well format, Panbio, MA, USA) according to manufacturer’s instructions. In brief, 100 µL of serum samples (10 µL serum diluted in 1000 µL serum diluent solution of the kit) was mixed with the monoclonal antibody tracer and the antigen diluent (DENV1-4 antigen pool) and incubated for 1 hr at 37 °C. After incubation the plate was washed 6 times followed by addition of 100 µL TMB solution. The reaction was stopped by addition of stop solution after ten minutes and the OD measured in 450 nm wavelength with a reference filter 600–650 nm. 

Viral RNA was isolated from the 140 µL of serum samples using a commercial viral RNA extraction kit QIAamp Viral RNA Mini Kit (Qiagen, Hilden, Germany) protocol and eluted in 60 µL of AVE elution buffer. An amount of 10µL of eluate was converted to cDNA using High-Capacity RNA-to-cDNA kit (Applied Biosystems, USA) following manufacturer’s instructions. Reverse transcription was performed at 37 °C for 60 minutes followed by 95 °C for 5 minutes using the random octamers. The reaction was set up in a 20 µL mastermix for cDNA conversion. 

The reverse transcriptase-polymerase chain reaction was performed using the primers and conditions as previously reported [18,19]. In brief, the cDNA was amplified with the primers amplifying the dengue virus capsid pre-membrane region spanning 511 bp sequences with a consensus forward-5′-TCAATATGCTGAAACGCGCGAGAAACC G-3′ and reverse 5′-TTGCACCAACAGTCAATGTCTTCAGGTTC-3′ primer for detection of dengue virus. For type discrimination, the consensus forward primer and four different reverse primers 5′-CGTCTCAGTGATCCGGGGG-3′ for the DENV-1 (482bp), 5′-CGCCACAAGGGCCATGAACAG-3′ for DENV-2 (119bp), 5′-TAACATCATCATGAGACAGAGC-3′ for DENV-3 (290bp), and 5′-CTCTGTTGTCTTAAACAAGAGA-3′, used for amplifying DENV-4 (392bp). The PCR master mix constitute of 10× PCR buffer, 1.5mM MgCl_2_, 200 µm of dNTPs, 1 unit of Taq DNA polymerase, 10 pmol primers in a 50 µL master mix run in PCR Mastercycler (Eppendorff, Hamburg, Germany) comprised of 40 cycles; with denaturation at 94 °C for 30 s, annealing at 52 °C for 30 s, extension at 72 °C for 30 s with initial denaturation at 94 °C for 5 min and final extension at 72 °C. Further type specific nested PCR was performed using the external product as template for serotype discrimination with an annealing temperature of 52 °C. The PCR products were resolved on 2% agarose gel and visualized with staining ethidium bromide. Selected amplicons were gel purified and sequenced using ABI genetic Analyzer and Big Dye Terminator DNA sequencing kit, to ascertain the findings (Applied Biosystems, Foster City, CA, USA) at the Institute of Life Sciences, core sequencing facility. The sequences were submitted to national center for biotechnology information (NCBI). 

### 2.3. Analyses of 41plex Cytokines Using Multiplex Luminex System

The levels of 41 cytokine molecules were measured using Luminex based bead array, the MILLIPLEX^®^ MAP Human Cytokine/Chemokine panel on LuminexxMAP^®^ platform using a magnetic bead format (MILLIPLEX^®^ Analytes, Millipore, MA, USA,) for the following biomarkers: EGF, FGF, Eotaxin, TGF-α, G-CSF, Flt, GM-CSF, Fractalkine, IFN-α2, IFN-γ, GRO, IL-10, MCP, IL-12P40, MDC, IL-12P70, IL-13, IL-15, sCD40L, IL-17A, IL-1RA, IL-1α, IL-9, IL-1β, IL-2, IL-3, IL-4, IL-5, IL-6, IL-7, IL-8, IP-10, MCP-1, MIP-1α, MIP-1β, TNF-α, TNF-β, VEGF, PDGF-AA, PDGF-BB, and RANTES. In brief, 25 µL plasma samples were tested for the levels of the cytokines, using the multiplex immunoassay containing fluorescent labeled beads conjugated with specific monoclonal antibody for the target molecule, according to the manufacturer’s recommendations [14,20]. Appropriate standards including 41 beads and quality control (provided in the kit) were run with the test sample plate. The software determines and interprets the data and concentrations of analytes in samples determined from the standards run in the test. Samples were considered for analysis using the standards that were run in each of the plates. Each run contained appropriate quality controls run in duplicates and results were interpreted from the standard using Bioplex 200 system (Bio-Rad, Hercules, CA, USA). 

### 2.4. Principal Component Analysis (PCA), Partial Least Squares Discriminant Analysis (PLS-DA)

MetaboAnalyst 4.0 software (available online: http://www.metaboanalyst.ca) for multivariate Principal Component Assay (PCA) and Partial Least Squares Discriminant Analysis (PLS-DA), based on R was used to comprehensively analyze the 41-plex cytokine bead array. Analysis of the 41-plex cytokine expression for different groups, i.e., dengue serotype, control vs dengue fever and dengue fever versus severe dengue by high dimensional spectral features using multivariate PCA and further discrimination of inter-class variance between the groups were made by the PLS-DA [21,22].The variable importance in the projection (VIP) score for the multiplex cytokine profiles in PLS-DA, that summarizes contributions of the most prominent cytokines contributing to the observed phenotypic variability is depicted in the model. 

### 2.5. Statistical Analysis 

Statistical analyses were performed using software GraphPad Prism® software PRISM 6 version, GraphPad Software, Inc., San Diego, CA, USA. The cytokines/chemokine data were analyzed using Student’s t test or ANOVA as appropriate. Fisher’s exact test was used for comparison of genotype, allele frequencies, and to test association of combined allele distribution among various clinical categories. Proportions were computed with 95% confidence intervals (95% C.I.) according to the Poisson distribution and a ‘p’ value less than 0.05 was considered as statistically significant. 

## 3. Results

### 3.1. Population Demographics

The demographic characteristics were as follows: age distribution ranged from 17 to 79 years, 34.2 ± 12.9 (mean ± SD). Males constituted 75% while the rest were females. When stratified for age, the majority of the subjects were in the 25–34 years age group. All subjects presented with a febrile condition. Subjects presented the illness for a duration of 4.3 ± 1.8 (SD) days. The baseline haemoglobin measured 11.7 ± 1.1 gms/dL, total leukocyte count 6419 ± 2280 cells/cubic millimetre of blood, platelet count 164 ± 685 ×10^3^/mm3, bilirubin in 0.37 ± 0.3, SGOT 101.9 ± 103.87 units/ml, SGPT in 75.2 ± 88.6 units/ml, urea 22.3 ± 9.4 mg /dL, serum creatinine measured 0.8 ± 0.2 mg/dL. Detailed clinical presentations in dengue and severe dengue are shown, Table S1. Among the clinical manifestations of retro-orbital pain and bleeding manifestations were statistically significant between the dengue fever (with and without warning signs) and severe dengue group (Tables S1 and S2). Based on the clinical conditions, subjects were treated by >1 of the authors, with conservative fluid management, prophylactic antibiotic treatments, and were considered for platelet transfusion based on disease severity as per the WHO guidelines.

### 3.2. Dengue Testing

Of the 98 subjects evaluated for the study, 85 (87%) [95% C.I: 78.48–92.22%] were found positive by NS1/IgM capture ELISA/RT-PCR during this outbreak. The thirteen subjects negative by NS1, IgM ELISA and negative by RT-PCR for DENV, were removed from analysis. Hence, further analysis for cytokine profiling was performed on the 85 dengue positive cases, and age and sex matched healthy controls. Among these 85 positive dengue cases, 73 (85.88%) of them presented with dengue fever (with and without warning signs) and 12 (14.11%) patients were categorized as severe dengue based on WHO criteria 2009 [16,17]. The cytokine profiling for those that were statistically significant were reported. 

Dengue serotype-specific RT-PCR discriminated for DENV in 47 (55.2%) patients; single infection for one serotype was observed in 61.7% while mixed infections with more than one serotype were detected in 38.29% patients. Overall there were 64 infections in 47 RT-PCR positive subjects for the four dengue serotypes -DENV-1 in 20%, DENV-3 in 17%, DENV-4 in 8% while the majority (55%) were positive for DENV-2 either as single or as multiple infections with other serotypes (Table 1). Sequencing by Sanger’s method confirmed the serotype finding by type-specific RT-PCR for single infections. 

### 3.3. Sequences and NCBI Accession Number

The sequences of representative circulating dengue strains submitted to NCBI (Accession number-MF069161, MF441493).

### 3.4. Multiplex Cytokine Bead Array (Inflammatory Markers as Predictors of The Dengue Severity) and Principal Component Assay 

**Figure 1:**
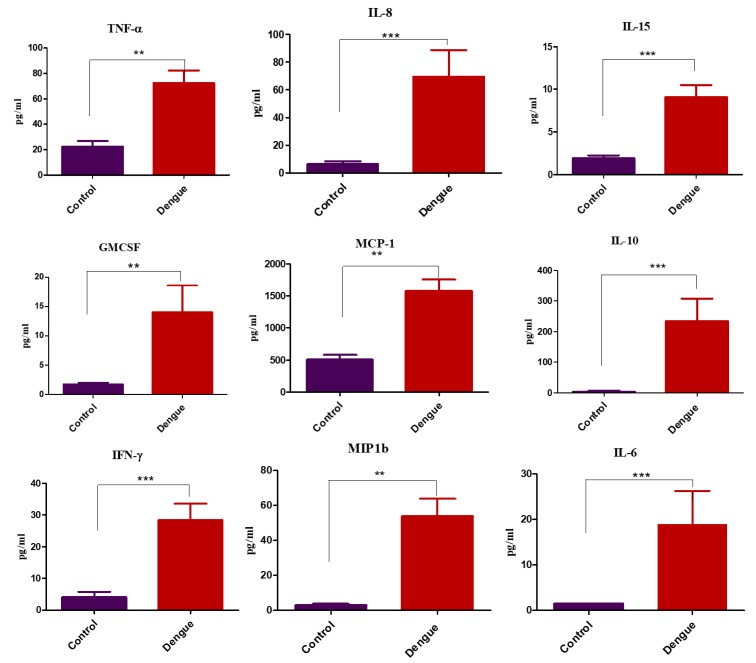
Cytokine levels in healthy controls and dengue positive subjects. Cytokine expression profile among the control vs dengue positive subjects. Of the 41 cytokine examined through Luminex bead array nine cytokines found to be associated with the dengue positivity when compared to the healthy controls. Statistical analyses were performed using Prism^®^ (version 6, GraphPad Software, CA, USA). Proportions were computed with the corresponding 95% confidence intervals (95% C.I.) according to the Poisson distribution. A ‘P’ value less than 0.05 was considered as statistically significant (* = *p* < 0.05, ** = *p* < 0.01 and *** = *p* < 0.001).

**Figure 2:**
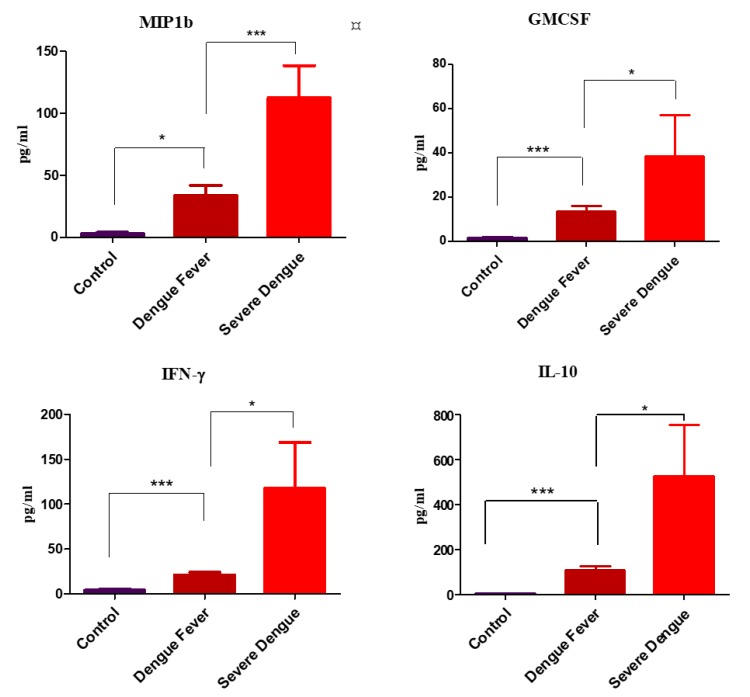
Cytokine levels in control, dengue fever (with and without warning signs) and in severe dengue. Cytokine expression profile among the control, mild dengue (with and without warning signs) and severe dengue subjects. Of the 41 cytokine examined through Luminex bead array, four cytokines were found to be associated with disease severity, when compared to the dengue patients; i.e., dengue fever (with and without warning signs), severe dengue, and the disease free controls. Statistical analyses were performed using Prism^®^ (version 6, GraphPad Software, CA, USA. A ‘P’ value less than 0.05 was considered as statistically significant (* = *p* < 0.05, ** = *p* < 0.01 and *** = *p* < 0.001). The cytokines IFN-γ, GM-CSF, IL-10 and MIP-1β could be the predictor of disease severity.

In dengue patients the nine plasma cytokine levels were significantly elevated when compared to healthy controls: GM-CSF, IFN-γ, IL-10, 1L-15, IL-8, MCP-1, IL-6, MIP1β, and TNFα (*p* = 0.008, 0.0001, 0.0001, 0.0003, 0.0001, 0.001, 0.0003, 0.001 and 0.006 respectively) levels (Figure 1). Increased plasma cytokine levels of IFN-γ, MIP-1β, GM-CSF and IL-10 and (*p* = 0.02, 0.0004, 0.03, 0.034) significantly differentiated severe dengue cases from patients with dengue fever in the cohort studied by us (Figure 2). 

### 3.5. Principal Component Assay (PCA), sparsePLS-DA & the variable’s importance VIP Score

**Figure 3:**
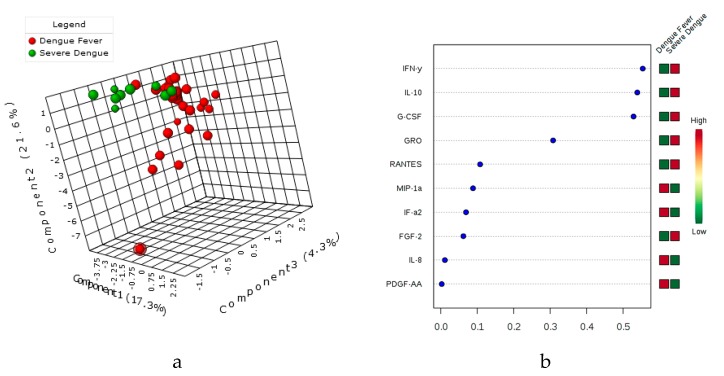
sPLS-DA and VIP score of Cytokine expression profile in Dengue fever vs Severe Dengue Patients. The Sparse Partial Least Squares Discriminant Analysis (sPLS-DA) depicting the cluster between the multiplex cytokine expression profile between the dengue fever (with and without warning signs) vs severe dengue patients (Figure 3a). The red circle represents for the multiplex cytokine expression for dengue fever, while the green color depicted for severe dengue patients (3b). The variable importance in the projection (VIP) score for the multiplex cytokine profile summarizes the contributions of the variables depicted in the model (3b). The expression of cytokines IFN-γ, IL-10, G-CSF, GRO, RANTES, and FGF-2 is higher in severe dengue compared to dengue fever patients.

**Figure 4:**
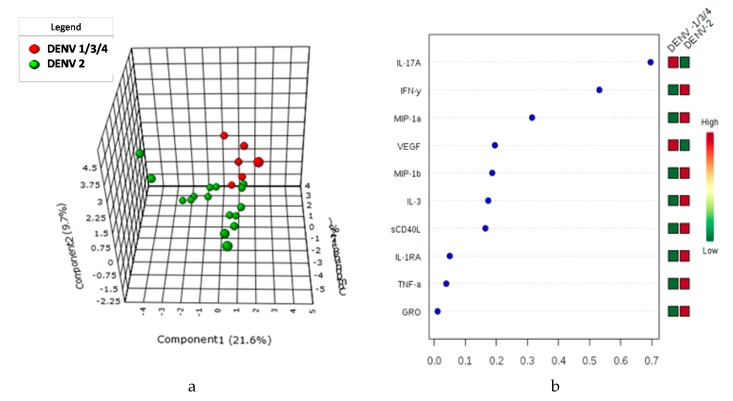
sPLS-DA and VIP score of Cytokine expression profile in DENV-2 vs DENV -1, -3, -4 serotypes in Dengue Patients. The Sparse Partial Least Squares Discriminant Analysis (sPLS-DA) depicting the cluster between the multiplex cytokine expression profile between the DENV-2 vs DENV-1, DENV-3 and DENV-4. The red circle represents for the DENV-1, -3, -4, while the green color depicted for DENV-2 subjects cluster (4a). The variable importance in the projection (VIP) score for the multiplex cytokine profile summarizes the contributions of the variables depicted in the model (4b). The DENV-2 vs DENV-1, -3, -4 group clustered distinctly in terms of the expression profile of the soluble biomarkers.

Multivariate analysis by Principal Component Assay, PLS-DA carried out for the 41 cytokine expression differentiated between dengue fever and severe dengue patients (Figure 3). Further for serotype discrimination in subjects with cytokine expression profile for DENV-2 from DENV-1, -3, -4 (pooled as a single group) for the biomarkers tested in this study (Figure 4). The variable importance in the projection (VIP) scores for the multiplex cytokine profiles in PLS-DA that summarize contributions of the signature cytokines for each group (Figure 3 and Figure 4) are depicted in the projection.

## 4. Discussion

One of the unsolved conundrum in Dengue pathogenesis is the consistent observation that a small proportion of dengue infected patients develop severe clinical manifestations. The factors that contribute to development of severe dengue has been a subject of intense study in recent years. Viral serotypes and differences host immune responses have been proposed as possible potential factors [4,11,23].

All four serotypes of DENV were detected in this outbreak in circulation, with DENV-2 (55%) as the predominant serotype in this region. The predominance of DENV-2 (also attributable fraction seen in single and mixed infections) in severe dengue subjects emphasizes the possible higher virulence of DENV-2 with disease severity in this outbreak. Several studies supported the role of DENV-2 serotypes in disease severity in other geographical regions; Molly OhAinle, et al. reported an abrupt increase in disease severity in dengue serotype 2 transmissions at Managua, Nicaragua (2011) [24]. In other study, Rico-Hesse R reported the associations of dengue serotype 2 viruses with the disease severity [25]. 

DENV is known to infect various immune cells including monocytes and dendritic cells, which majorly contributes to production of inflammatory and/or antiviral cytokines. Unregulated inflammation leads to exacerbated pathogenesis and tissue/organ injuries followed by death [12,26]. Since patients with dengue are characterized by excessive secretion of cytokines and chemokines, analysis of cytokine and chemokine levels could be a tool for predicting status/severity of the disease. 

In this study, the multiplex bead array reported for the first time literature in human patients infected with dengue virus revealed that the GM-CSF, IFN-γ, IL-10, IL-15, IL-8, MCP-1, IL-6, MIP-1β, and TNF-α levels were significantly raised in dengue subjects compared to healthy controls. Our finding is consistent with other studies in association of IL-6, IL-8, IL-15, MCP-1, and TNF-α in dengue fever [4,10,27,28]. However, the most critical observation was that IFN-γ, GM-CSF, IL-10, and MIP-1β differentiated severe dengue patients from dengue fever cases in our cohort. While similar observations have been made by others for IFN-γ and IL-10 in severe dengue [10,29,30,31,32], increased levels of GM-CSF and MIP-1β novel to the present study. A recent report from GWAS studies on asymptomatic and clinical dengue subjects revealed most of the changes seen in expression profile belong to immune responses [33]. However, this study examined the transcriptome profile (rather than plasma levels) in patients with and without symptomatic dengue. The induction of pro-inflammatory molecules IFN-γ and GM-CSF through induction of effector molecules may lead to vascular permeability and contribute in pathogenesis [34]. Macrophage inflammatory protein-1 beta (MIP-1b), the chemoattractant produced by the monocytes, dendritic cells, NK cells recruits the immune cells at the site of inflammation [35]. The monocyte chemotactic protein-1 (MCP-1) is a potent chemotactic factor that regulates migration of monocytes/macrophages. Further this migration across the vascular endothelium is in response to dengue viral infection leads to change in permeability in vascular endothelium through reduced tight junctions [36,37,38]. In addition, our study also reported the association of GM-CSF and MIP-1β as biomarkers in disease severity. This finding is consistent with a recent report by Bozza et al., which examined seventeen cytokines for the predictor of disease severity, found to be MIP-1β and IFN-γ as predictors of disease severity. 

Thus levels of these plasma cytokines could be used as biomarkers of both severity and possibly for prognosis and their antagonists could be potentially be used as therapeutic agents. More interestingly, multivariate analysis by PCA, PLS-DA and VIP score also effectively discriminated DENV-2 from DENV-1, -3, -4 (pooled as a single group) for the inflammatory biomarkers. The current study demonstrates involvement of DENV serotype-2 association with disease severity accompanied by elevated plasma inflammatory mediators and thus underscores the interplay of serotype-specific immune response in determination of dengue disease severity. A similar report on the disease severity by the dengue virus serotype-2 was demonstrated in Nicaragua with serotype specific immunity [24,25].

The data presented in this communication however have limitations, since the numbers of cases with severe dengue was not high during this outbreak. However, it is unlikely to have any effect on the broad findings of the key molecules in terms of the data outcome. Adequate age and sex matched controls were included for the detection and used in the multiplex bead array for measuring the inflammatory molecules. Besides conducting a luminex assay for 41 host molecules in circulation, all samples were analyzed by multiple methods for diagnosis and multivariate analysis computed through the PLS-DA, sparse discriminant analysis through sPLS-DA. Further we have used the dimension reduction by global analysis for the 41 plex cytokines vs different clinical conditions and against different serotypes using PCA and the PLS-DA based R program to compensate for the study subject numbers in severe dengue [22,39]. Since the number of viral serotypes DENV-1, DENV-3, and DENV-4 are less in number compared to DENV-2, we did the dimension reduction analysis for DENV-2 vs other DENV serotypes. Screening a much larger cohort of patients may have contributed to higher stringency for analysis of frequency of each of the four viral serotypes and levels of inflammatory molecules in Dengue patients. The epidemiology of DENV is constantly changing. An effective vaccine for dengue is now a distinct possibility in the foreseeable future. Indeed, we need more data and corroborating host immune response and serotype information, and this study would be valuable in designing optimum intervention strategies. 

In summary, this report demonstrates the predominance of DENV serotype 2 in current outbreak and infection with multiple serotypes found in our study. Further, serotype-2 is predominant in severe dengue cases and marked by elevated expression of inflammatory molecules. This study reports the key inflammatory mediators IFN-γ, GM-CSF, IL-10, and MIP-1β as markers of disease severity in dengue patients. This information is of value and could be utilized for further studies for translational potential of these molecules and help guide in optimizing therapeutic efforts.

## Figures and Tables

**Table 1:** Circulation of dengue serotypes in 2016 outbreak in eastern India.

Dengue Serotype *N* = 47	Frequency (%)
*Single Infection (N = 29)*	
DENV-1	1 (3.44)
DENV-2	20 (68.86)
DENV-3	7 (24.13)
DENV-4	1 (3.44)
*Mixed infections (N = 18)*	
DENV-1 & -2	9 (50)
DENV-1 & -4	3 (16.66)
DENV-2 & -3	5 (27.77)
DENV-2 & -4	1 (5.55)

Typing of dengue positive patients by type specific RT-PCR (*N* = 47). Single infection seen in 29 cases, while mixed infections were detected in 18 cases. The percentage shown, are calculated for single and mixed infections separately.

## Supplementary Materials

Additional data available in Supplement section. The following are available online at http://www.mdpi.com/1999-4915/11/1/34/s1

## Presented in Part

International Vaccine Conference, 27–29 Nov 2017, ICGEB, New Delhi, India; First joint American Society for Cell Biology (ASCB) and European Molecular Biology Organization (EMBO) 2017 Annual Meeting, Pennsylvania Convention Center, Philadelphia, PA, USA, 2–6 December 2017.

## References

[1] Bhatt S., Gething P.W., Brady O.J., Messina J.P., Farlow A.W., Moyes C.L., Drake J.M., Brownstein J.S., Hoen A.G., Sankoh O. (2013). The global distribution and burden of dengue. Nature.

[2] Lindenbach B.D., Murray C.L., Thiel H.J., Rice C.M., Knipe D.M., Howley P.M. (2013). Flaviviridiae. Fields Virology.

[3] Halstead S.B. (1988). Pathogenesis of dengue: Challenges to molecular biology. Science.

[4] Rothman A.L. (2011). Immunity to dengue virus: A tale of original antigenic sin and tropical cytokine storms. Nat. Rev. Immunol..

[5] Screaton G., Mongkolsapaya J., Yacoub S., Roberts C. (2015). New insights into the immunopathology and control of dengue virus infection. Nat. Rev. Immunol..

[6] Waggoner J.J., Balmaseda A., Gresh L., Sahoo M.K., Montoya M., Wang C., Abeynayake J., Kuan G., Pinsky B.A., Harris E. (2016). Homotypic Dengue Virus Reinfections in Nicaraguan Children. J. Infect. Dis..

[7] Halstead S.B. (2007). Dengue. Lancet.

[8] Guzman M.G., Harris E. (2015). Dengue. Lancet.

[9] Katzelnick L.C., Gresh L., Halloran M.E., Mercado J.C., Kuan G., Gordon A., Balmaseda A., Harris E. (2017). Antibody-dependent enhancement of severe dengue disease in humans. Science.

[10] Bozza F.A., Cruz O.G., Zagne S.M., Azeredo E.L., Nogueira R.M., Assis E.F., Bozza P.T., Kubelka C.F. (2008). Multiplex cytokine profile from dengue patients: MIP-1beta and IFN-gamma as predictive factors for severity. BMC Infect. Dis..

[11] Soo K.M., Khalid B., Ching S.M., Tham C.L., Basir R., Chee H.Y. (2017). Meta-analysis of biomarkers for severe dengue infections. PeerJ.

[12] Costa V.V., Fagundes C.T., Souza D.G., Teixeira M.M. (2013). Inflammatory and innate immune responses in dengue infection: Protection versus disease induction. Am. J. Pathol..

[13] Rathakrishnan A., Wang S.M., Hu Y., Khan A.M., Ponnampalavanar S., Lum L.C., Manikam R., Sekaran S.D. (2012). Cytokine expression profile of dengue patients at different phases of illness. PLoS ONE.

[14] Won J.H., Goldberger O., Shen-Orr S.S., Davis M.M., Olshen R.A. (2012). Significance analysis of xMap cytokine bead arrays. Proc. Natl. Acad. Sci. USA.

[15] WHO Recommendations of the Strategic Advisory Group of Experts on Immunization (SAGE) on the Use of Dengvaxia. http://www.who.int/immunization/diseases/dengue/revised_SAGE_recommendations_dengue_vaccines_apr2018/en/.

[16] WHO/TDR (2009). Dengue: Guidelines for Diagnosis, Treatment, Prevention and Control.

[17] Alexander N., Balmaseda A., Coelho I.C., Dimaano E., Hien T.T., Hung N.T., Janisch T., Kroeger A., Lum L.C., Martinez E. (2011). Multicentre prospective study on dengue classification in four South-east Asian and three Latin American countries. Trop. Med. Int. Health.

[18] Lanciotti R.S., Calisher C.H., Gubler D.J., Chang G.J., Vorndam A.V. (1992). Rapid detection and typing of dengue viruses from clinical samples by using reverse transcriptase-polymerase chain reaction. J. Clin. Microbiol..

[19] Saswat T., Kumar A., Kumar S., Mamidi P., Muduli S., Debata N.K., Pal N.S., Pratheek B.M., Chattopadhyay S. (2015). High rates of co-infection of Dengue and Chikungunya virus in Odisha and Maharashtra, India during 2013. Infect. Genet. Evol..

[20] Patro A.R.K., Prusty B.K., Gaikwad S., Singh D.K., Mohanty S., Das B.K., Ravindran B. Identification of inflammatory biomarkers in dengue disease severity in eastern India. Proceedings of the Joint American Society for Cell Biology (ASCB) and European Molecular Biology Organization (EMBO) 2017 Annual Meeting, Pennsylvania Convention Center.

[21] Xia J., Wishart D.S. (2016). Using MetaboAnalyst 3.0 for Comprehensive Metabolomics Data Analysis. Curr. Protoc. Bioinform..

[22] Chong J., Soufan O., Li C., Caraus I., Li S., Bourque G., Wishart D.S., Xia J. (2018). MetaboAnalyst 4.0: Towards more transparent and integrative metabolomics analysis. Nucleic Acids Res..

[23] Vaughn D.W., Green S., Kalayanarooj S., Innis B.L., Nimmannitya S., Suntayakorn S., Endy T.P., Raengsakulrach B., Rothman A.L., Ennis F.A., Nisalak A. (2000). Dengue viremia titer, antibody response pattern, and virus serotype correlate with disease severity. J. Infect. Dis..

[24] OhAinle M., Balmaseda A., Macalalad A.R., Tellez Y., Zody M.C., Saborío S., Nuñez A., Lennon N.J., Birren B.W., Gordon A. (2011). Dynamics of Dengue Disease Severity Determined by the Interplay Between Viral Genetics and Serotype-Specific Immunity. Sci. Transl. Med..

[25] Rico-Hesse R., Harrison L.M., Salas R.A., Tovar D., Nisalak A., Ramos C., Boshell J., de Mesa M.T., Nogueira R.M., da Rosa A.T. (1997). Origins of dengue type 2 viruses associated with increased pathogenicity in the Americas. Virology.

[26] Srikiatkhachorn A., Mathew A., Rothman A.L. (2017). Immune-mediated cytokine storm and its role in severe dengue. Semin. Immunopathol..

[27] Green S., Vaughn D.W., Kalayanarooj S., Nimmannitya S., Suntayakorn S., Nisalak A., Rothman A.L., Ennis F.A. (1999). Elevated plasma interleukin-10 levels in acute dengue correlate with disease severity. J. Med. Virol..

[28] Butthep P., Chunhakan S., Yoksan S., Tangnararatchakit K., Chuansumrit A. (2012). Alteration of cytokines and chemokines during febrile episodes associated with endothelial cell damage and plasma leakage in dengue hemorrhagic fever. Pediatr. Infect. Dis. J..

[29] Nguyen T.H., Lei H.Y., Nguyen T.L., Lin Y.S., Huang K.J., Le B.L., Lin C.F., Yeh T.M., Do Q.H., Vu T.Q. (2004). Dengue hemorrhagic fever in infants: A study of clinical and cytokine profiles. J. Infect. Dis..

[30] Priyadarshini D., Gadia R.R., Tripathy A., Gurukumar K.R., Bhagat A., Patwardhan S., Mokashi N., Vaidya D., Shah P.S., Cecilia D. (2010). Clinical findings and pro-inflammatory cytokines in dengue patients in Western India: A facility-based study. PLoS ONE.

[31] Malavige G.N., Gomes L., Alles L., Chang T., Salimi M., Fernando S., Nanayakkara K.D., Jayaratne S., Ogg G.S. (2013). Serum IL-10 as a marker of severe dengue infection. BMC Infect. Dis..

[32] Lee Y.H., Leong W.Y., Wilder-Smith A. (2016). Markers of dengue severity: A systematic review of cytokines and chemokines. J. Gen. Virol..

[33] Simon-Loriere E., Duong V., Tawfik A., Ung S., Ly S., Casademont I., Prot M., Courtejoie N., Bleakley K., Buchy P. (2017). Increased adaptive immune responses and proper feedback regulation protect against clinical dengue. Sci. Transl. Med..

[34] Kurane I., Innis B.L., Nisalak A., Hoke C., Nimmannitya S., Meager A., Ennis F.A. (1989). Human T cell responses to dengue virus antigens. Proliferative responses and interferon gamma production. J. Clin. Investig..

[35] Spain-Santana T.A., Marglin S., Ennis F.A., Rothman A.L. (2001). MIP-1 alpha and MIP-1 beta induction by dengue virus. J. Med. Virol..

[36] Taub D.D., Proost P., Murphy W.J., Anver M., Longo D.L., van Damme J., Oppenheim J.J. (1995). Monocyte chemotactic protein-1 (MCP-1), -2, and -3 are chemotactic for human T lymphocytes. J. Clin. Investig..

[37] Gu L., Rutledge B., Fiorillo J., Ernst C., Grewal I., Flavell R., Gladue R., Rollins B. (1997). In vivo properties of monocyte chemoattractant protein-1. J. Leukoc. Biol..

[38] Lee Y.R., Liu M.T., Lei H.Y., Liu C.C., Wu J.M., Tung Y.C., Lin Y.S., Yeh T.M., Chen S.H., Liu H.S. (2006). MCP-1, a highly expressed chemokine in dengue haemorrhagic fever/dengue shock syndrome patients, may cause permeability change, possibly through reduced tight junctions of vascular endothelium cells. J. Gen. Virol..

[39] Lê Cao K.A., Boitard S., Besse P. (2011). Sparse PLS discriminant analysis: Biologically relevant feature selection and graphical displays for multiclass problems. BMC Bioinform..

