# Supplementary material for: Cytokine Signature Associated with Disease Severity in Dengue
Source: Viruses. 2019 Jan 8;11(1):34. doi: 10.3390/v11010034 (PMC6357178; doi:10.3390/v11010034)
Supplement: Supplementary file 1 [file viruses-11-00034-s001.pdf]

Supplementary Information

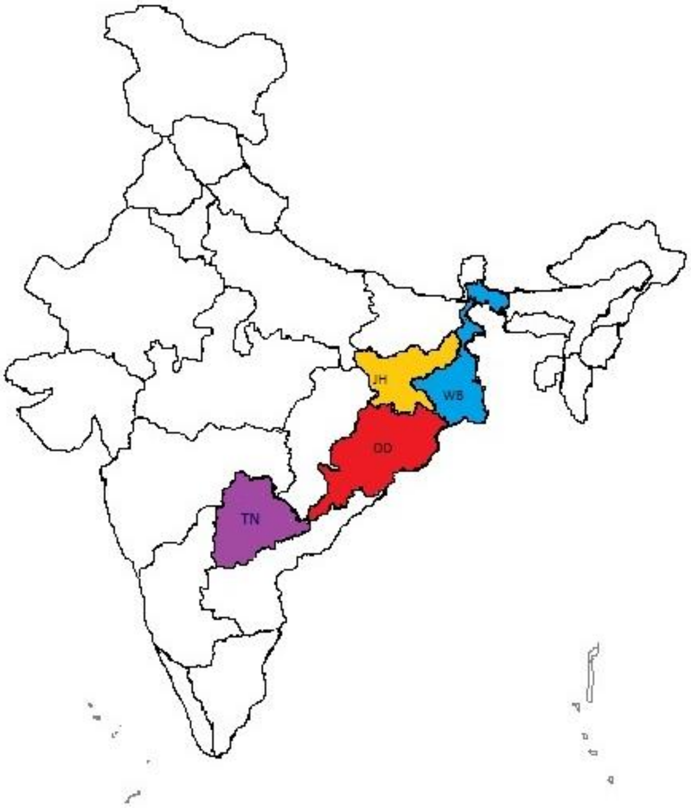

Figure S1. Map of the States and location from where the subjects were presented with Dengue 2016, India. **Map of India**. Detail map of India showing the states from where the dengue samples were collected. OD= Odisha (Red), TN= Telengana (purple), JH= Jharkhand Yellow) , WB= West Bengal (Blue) [India is located in Northern hemisphere between 8°4′N to 37°6′ north latitudes and 68°7′E and 97°25′east longitudes with the Tropic of Cancer cuts the landmass half way through at 23°30′ north latitude].

Table S1. Comparison of clinical presentation in dengue fever (with & without warning Signs) and severe dengue patients (N = 85).

Clinical Presentation	Dengue Fever No. (%) (n = 73)	Severe Dengue No. (%) (n = 12)	*p*-value *
Fever	73 (100%)	12 (100%)	1.00
Headache	68 (93.15%)	9 (75 %)	0.08
Myalgia	54 (73.97%)	6 (50 %)	0.16
Arthralgia	48 (65.75%)	5 (41.66 %)	0.12
Dizziness	28 (38.35%)	6 (50%)	0.53
Bodyache	55 (75.34%)	6 (50 %)	0.08
Nausea	35 (47.94%)	5 (41.66 %)	0.76
Vomitting	23 (31.50%)	6 (50%)	0.32
Retroorbital pain	15 (20.54%)	7 (58.33 %)	0.03
Skin Rash	17 (23.28%)	4 (33.33%)	0.47
Itching	18 (21.2%)	2 (16.66%)	0.72
Abdominal pain	8 (10.95%)	4 (33.33%)	0.06
Loose motion	26 (35.61%)	4 (33.33%)	1.00
Oliguria	8 (10.95%)	3 (25%)	0.18
Bleeding Manifestation	0	8 (66.6 %)	0.0001

* *p* value (Fisher's exact test, two tailed)

Table S2. Characteristic of clinical presentation and serotypes in subjects with severe dengue (*N* = 12).

Subject	Gender	Age	Duration of fever (days)	Dengue Serotype	Platelet count (cells ×10^3/mm^3	Bleeding Manifestation	Complications if any	Outcome
1	M	20	5	2	1.5	No	Pleural effusion	Improved
2	F	25	5	2, 3	1.0	Yes	Blanching erythema, Conjunctional suffusion, skin rash	Death
3	M	28	4	2	2.0	Yes	Melena	Improved
4	M	26	5	2	1.2	No	Hepatopathy	Improved
5	F	47	3	2	1.6	Yes	Oliguria, Hepatomegaly Pleural effusion	Improved
6	M	35	3	3	0.4	Yes	Skin Rash	Improved
7	M	34	6	2	0.6	Yes	Hematuria	Improved
8	F	28	5	1, 2	2.0	Yes	Oliguria	Improved
9	M	39	3	3	1.0	No	Melena, Conjunctional suffusion,	Improved
10	F	41	3	2, 4	0.83	No	Skin Rash, Pleural effusion	Improved
11	M	28	5	2	1.2	No	Hepatopathy	Improved
12	F	22	2	2	0.44	Yes	Melena	Improved

M= Male, F = Female

Table S3. Key Resources Table. List of Chemicals, reagents, kits, scientific instruments-manufacturer and software used in this study.

Name of the Chemical, Reagent	Details/ Catalog Number	Make
K2-EDTA anticoagulant vacutainer- 4 ml	367844	BD Biosciences
Serum Tube with Clot Activator- 4ml	367812 BD	BD Biosciences
DNase, RNase, and Pyrogen Free Plastic ware-Aerosol Filter Tips, Sterile, 0.5 ml, 1.5 ml, 2 ml, Maxy clear sterile tubes		Axygen
RNaseZap RNase Decontamination Solution	AM9780	Invitrogen
Dengue NS1, IgG,	N/A	Avantor
Dengue IgM Capture ELISA kit	01PE20	PANBIO
UltraPure DNase/RNase-Free Distilled Water	10977035	Invitrogen
DNase I	18047019	Invitrogen
2-Propanol Mol biol (500 ml)	I9516	Sigma
PCR Tubes with Flat Cap, 0.2mL, RNase, DNase, pyrogen-free.	PCR-05-L-C	Axygen
QIAamp Viral RNA Mini Kit (250 rxn)	52906	QIAGEN
Ethanol (500 ml)	100983	Merck
High-Capacity RNA-to-cDNA Kit	4387406	Applied Biosystems
Oligos	N/A	IDT/ Eurofin
Taq DNA Polymerase GoTaq 5U/µl (500 U)	M3005	Promega
dNTPS mix (1000 µl)	U1515	Promega
6x Loading Dye	G1881	Promega
UltraPure Agarose	16500500	Invitrogen
UltraPure Ethidium Bromide, 10 mg/mL	15585011	Invitrogen
40x TAE (1000 ml)	V4281	Promega
100 bp DNA Ladder, ready-to-use 50 µg	SM0244	Thermo Scientific
QIAquick PCR Purification Kit (250)	28106	QIAGEN
QIAquick Gel Extraction Kit (250)	28706	QIAGEN
AmpliTaq Gold DNA Polymerase with Gold Buffer and MgCl2 (250 U) 5U/µl	4311814	Applied Biosystems
dNTP Mix (10 mM ea)	18427088	Applied Biosystems
BigDye Terminator v3.1 Cycle Sequencing Kit (100 rxn)	4337455	Applied Biosystems
MultiScreen BV Filter Plate, bead based assay 1.2 µm	MSBVN1210	Merck Millipore
Sealing Foil	04729757001	Roche
MILLIPLEX MAP Human Cytokine/Chemokine Magnetic Bead Panel - Premixed 41 Plex - Immunology Multiplex Assay	HCYTMAG-60K-PX41	Merck Millipore
Sheath Fluid	342003	BD Biosciences
Phosphate buffered saline	10010023	Gibco
Equipment		
NanoDrop 2000/2000c Spectrophotometers	ND2000LAPTOP	Thermo Scientific
Synergy UV-water purification system [18.2MΩ.cm]		Merck Millipore
ChemiDoc Imaging Systems		Bio-Rad
BIO-PLEX 200 System	Luminex X-MAS Technology	Bio-Rad
PowerPa Basic Power Supply [300V/400mA/75w]		Bio-Rad
Mini-Sub® Cell GT Cell		Bio-Rad
PCR Mastercycler Eppendorf AG22331		Eppendorf
Centrifuge 5810/ 5810 R		Eppendorf
Centrifuge Heraeus Centrifuge FRESCO21		Thermo
GeneAmp PCR system thermal cycler		Applied Biosystems
Automated DNA Sequencer ABI	ABI 3500	Applied Biosystems

Software

Name of the Software	version/ URL link
Multivariate Data Analysis Methods - Software:	PCA, PLS-DA, 3-D Plot- VIP Score<br>Metaboanalyst software version 4<br>http://www.metaboanalyst.ca/<br><br>Ref:<br>Chong, J.; Soufan, O.; Li, C.; Caraus, I.; Li, S.; Bourque, G.; Wishart, D. S.; Xia, J., MetaboAnalyst 4.0: towards more transparent and integrative metabolomics analysis. Nucleic Acids Res. 2018, 46, (W1), W486-W494.

References & URL Links:

Detail guidelines for diagnosis, subject inclusion, exclusion, detection methods, treatment, prevention and control [WHO]
DENGUE: Guidelines for Diagnosis, Treatment, Prevention and Control (2009) WHO. ISBN: 9789241547871; https://www.who.int/tdr/publications/documents/dengue-diagnosis.pdf

Clinical and Laboratory Guidelines for Dengue

https://www.cdc.gov/dengue/clinicallab/index.html

For preparation of buffer, common laboratory reagents & protocols:

Molecular Cloning: A Laboratory Manual, Vol I, II, III. (Fourth Edition); (2012) ISBN 978-1-936113-42-2; By Michael R. Green; Joseph Sambrook, Cold Spring Harbor Laboratory Press, USA

PANBIO Dengue IgM Capture ELISA

https://www.alere.com/en/home/product-details/panbio-dengue-igm-capture-elisa.html

WHO evaluation of ELISA kit Panbio

https://www.who.int/tdr/publications/documents/diagnostics-evaluation-3.pdf

DENV Detect™IgG ELISA Kit, InBios

http://www.inbios.com/denv-detect-igg-elisa/

DENV Detect™ NS1 ELISA Kit, InBios

http://www.inbios.com/denv-detecttm-ns1-elisa-kit-intl/

Viral RNA Extraction kit (Catalog number- 52906, Qiagen)

https://www.qiagen.com/us/resources/resourcedetail?id=c80685c0-4103-49ea-aa72-8989420e3018&lang=en

High-Capacity RNA-to-cDNA Kit

https://assets.thermofisher.com/TFS-Assets/LSG/manuals/MAN0017979_RNAtocDNA_QR.pdf
QIAquick PCR Purification Kit & QIAquick Gel Extraction Kit
https://www.qiagen.com/us/resources/resourcedetail?id=e0fab087-ea52-4c16-b79f-c224bf760c39&lang=en
MILLIPLEX MAP Human Cytokine/Chemokine Magnetic Bead Panel - Premixed 41 Plex - Immunology Multiplex Assay [HCYTMAG-60K-PX41], Merck, Millipore

http://www.merckmillipore.com/IN/en/product/MILLIPLEX-MAP-Human-Cytokine-Chemokine-Magnetic-Bead-Panel-Immunology-Multiplex-Assay,MM_NF-HCYTOMAG-60K?ReferrerURL=https%3A%2F%2Fwww.google.co.in%2F

For analysis of Bioplex data:

http://www.bio-rad.com/en-us/product/bio-plex-manager-software-standard-edition?ID=5846e84e-03a7-4599-a8ae-7ba5dd2c7684

http://www.bio-rad.com/en-us/applications-technologies/bio-plex-data-analysis-software?ID=M15GLB15
